# Assessment of Flurbiprofen Suspension and Composite Gel Pre- and Post Skin Perforation: Effectiveness in Managing Inflammatory Responses in Ear Tags and Periocular Piercings

**DOI:** 10.3390/gels11040292

**Published:** 2025-04-15

**Authors:** Sheimah El Bejjaji, Gladys Ramos-Yacasi, Valeri Domínguez-Villegas, Délia Chaves Moreira Dos Santos, Antonio Braza, Lilian Sosa, Maria José Rodríguez-Lagunas, Ana Cristina Calpena, Mireia Zelaya, Alexander Parra

**Affiliations:** 1 Department of Pharmacy, Pharmaceutical Technology and Physical Chemistry, Faculty of Pharmacy and Food Sciences, University of Barcelona, 08028 Barcelona, Spain; shelbejb7@alumnes.ub.edu (S.E.B.); drecoar@gmail.com (A.P.); 2 Facultad de Ciencias Farmacéuticas, Bioquímicas y Biotecnológicas, Universidad Católica de Santa María (UCSM), Arequipa 04001, Peru; glramos011@hotmail.es; 3 Facultad de Ciencias Químicas e Ingeniería, Universidad Autónoma del Estado de Morelos (UAEM), Cuernavaca, Morelos 62209, Mexico; valeri.dominguez@uaem.mx; 4 Department of Pharmacy and Nutrition, Federal University of Espírito Santo (UFES), Goiabeiras, Vitória 29075-910, Brazil; deliachavesmoreira@gmail.com; 5 Centro Experimental en Biociencia (CENBIO), Facultad de Ciencias Químicas y Farmacia, Universidad Nacional Autónoma de Honduras (UNAH), Tegucigalpa 11101, Honduras; liliansosa2012@gmail.com; 6 Instituto de Investigaciones en Microbiología (IIM), Facultad de Ciencias, Universidad Nacional Autónoma de Honduras (UNAH), Tegucigalpa 11101, Honduras; 7 Department of Biochemistry and Physiology, Faculty of Pharmacy and Food Science, University of Barcelona (UB), 08028 Barcelona, Spain; mjrodriguez@ub.edu; 8 Laboratory of Plant and Animal Histology, School of Biology, Faculty of Sciences, National Autonomous University of Honduras (UNAH), Tegucigalpa 11101, Honduras; mireya.zelaya@unah.edu.hn

**Keywords:** flurbiprofen, suspension, hydrogel, polyethylene glycol 3350, human skin, porcine skin, NSAID, transdermal drug delivery, controlled skin perforation, inflammation management

## Abstract

(1) Background: Controlled skin perforations, such as ear tags, piercings, and microdermal implants, induce inflammation and stress in individuals undergoing these procedures. This localized trauma requires care to optimize healing, reduce inflammation, and prevent infections. (2) Methods: Two formulations were developed: an FB-suspension and an FB-gel. Their in vivo efficacy was evaluated, along with drug retention in porcine and human skin after 30 min of administration, chemical stability at different temperatures, cytotoxicity, histological changes induced via transdermal application, and irritative potential, assessed using the HET-CAM assay. (3) Results: Both formulations reduced inflammation when applied 30 min before perforation compared to the positive control. The FB-suspension demonstrated no cytotoxicity and exhibited greater efficacy than the free flurbiprofen solution, highlighting the advantages of using nanoparticle-mediated drug delivery. Moreover, the FB-gel maintained chemical stability for up to 3 months across a temperature range of 4 to 40 °C. Histologically, no significant changes in skin composition were observed. (4) Conclusions: The FB-suspension is viable for both pre- and post-perforation application, as it is a sterile formulation. In contrast, the FB-gel is a convenient and easy application, making it a practical alternative for use in both clinical and veterinary settings.

## 1. Introduction

Identification ear tags serve as perforated attachments that enable individual animal identification, supporting traceability and compliance with sanitary and commercial regulations throughout an animal’s life cycle from birth to slaughter or market [1,2]. Many countries mandate the use of these tags to meet regulatory requirements for animal welfare and to effectively monitor livestock movements and commercial transactions.

The ear tagging procedure for pigs requires a specialized applicator that pierces the ear and fastens the tag securely. The process begins with cleaning the intended application site to reduce the infec tion risk. The operator then positions the loaded applicator in the central portion of the ear, carefully avoiding major vascular structures, before applying firm pressure to perforate the tissue and secure the identification device. Ensuring proper attachment and readability is critical, as these tags provide essential information required for herd management and traceability protocols.

Researchers have investigated the impact of ear tagging on tissue integrity, emphasizing that factors such as the animal’s age at tagging, environmental housing conditions, and precise tag placement play crucial roles in the risk of lesion development. Studies have underscored the importance of conducting ear examinations approximately two weeks after tagging to detect and address potential tissue complications before they escalate into more severe conditions, ultimately improving overall animal welfare. Furthermore, research findings have indicated that the tagging process triggers measurable stress responses and discomfort, highlighting the need for improved application techniques and exploring alternative identification methods.

In a related work, Barz and colleagues [3] studied how combined meloxicam and iron administration affects piglets undergoing castration. Their results indicate that meloxicam administration meaningfully reduces pain indicators and stress responses following the procedure, with positive implications for animal welfare standards. Further investigations remain essential to fully characterize the pathophysiological mechanisms underlying ear necrosis due to ear tagging and to develop targeted preventative interventions [4,5,6].

On the other hand, skin perforations in humans vary significantly. Unlike identification ear tags, whose perforation location is standardized, piercings can be applied to various areas of the body, leading to different healing processes depending on the pierced area. In this case, piercings serve esthetic and cultural purposes. The piercing process involves creating a controlled wound in the skin or cartilage using a sterile needle or catheter, followed by inserting a biocompatible piece of jewelry. Another method of inserting jewelry into human skin is via microdermal implants or dermal punches. The main difference from piercings is that piercings have both an entry and an exit point, whereas microdermal implants are anchored beneath the epidermis [7,8].

This study analyzes and compares the efficacy of two formulations characterized by El Bejjaji et al. [9] and Ramos et al. [10,11], designed for both preventing and treating inflammation associated with skin perforation procedures, such as ear tagging, piercings, or microdermal implants. To optimize their effectiveness and ensure user comfort, the formulations were administered 30 min before the procedure. This carefully planned timeframe was an essential component of the experimental design, allowing for thoroughly evaluating their anti-inflammatory properties in both preventative and therapeutic contexts.

These formulations contain flurbiprofen (FB), a nonsteroidal water-insoluble anti-inflammatory drug (NSAID) widely used in clinical practice for treating pain and inflammation, as the active ingredient. FB is encapsulated in freeze-dried polymeric nanoparticles of poly(ε-caprolactone) (*Pε*CL), incorporating polyethylene glycol 3350 (PEG) as a cryoprotectant and being sterilized using gamma (γ) irradiation.

**Figure 1:**
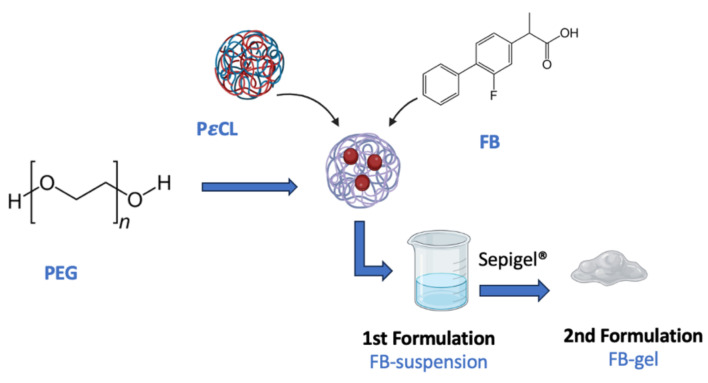
Preparing the formulations under study.

The first formulation corresponds to the suspension described by Ramos et al. and is sterile [9] and Ramos et al. [10,11]. The second formulation is obtained by incorporating the suspension into a hydrogel (Sepigel^®^), following the procedure described by El Bejjaji et al. [9]. A graphical representation of the study’s formulations is shown in Figure 1.

The selected active ingredient, FB, functions by inhibiting cyclooxygenase (COX) enzymes 1 and 2. COX-1 maintains constant levels in most tissues and facilitates the physiological production of prostaglandins. Its metabolites from arachidonic acid regulate critical functions, including gastrointestinal protection, vascular homeostasis, renal hemodynamics, and platelet activity [12]. In contrast, COX-2 appears primarily at inflammatory sites, in neoplasms, or under specific physiological conditions. This enzyme primarily synthesizes prostaglandins involved in inflammatory, pathological, and stress responses. Pro-inflammatory factors can induce its expression, potentially increasing the baseline concentration by 10 to 80 times [13].

FB demonstrates stronger inhibitory effects on the COX-1 enzyme, which accounts for its adverse effects, particularly in the gastrointestinal system. With a relatively brief half-life of approximately 4 h, FB requires frequent administration [14,15]. These limitations highlight the need to develop pharmaceutical formulations that bypass the gastrointestinal tract, such as dermal and transdermal delivery systems.

**Figure 2:**
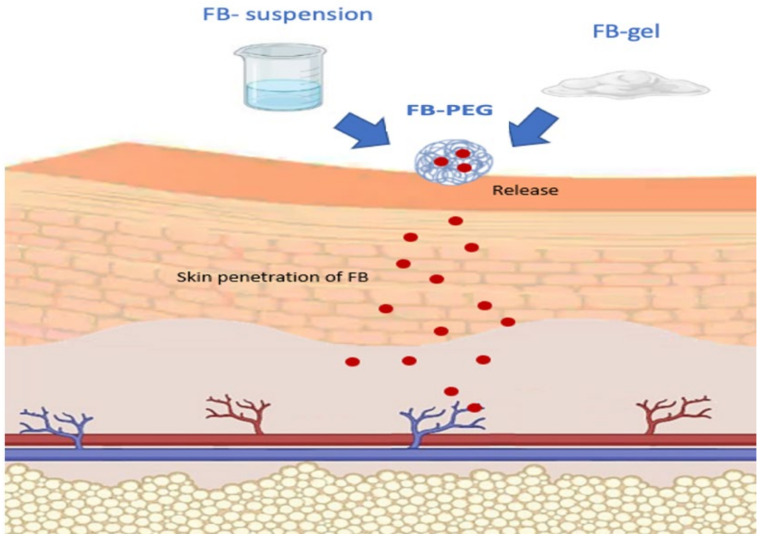
Flurbiprofen release process (red spheres) from nanoparticles.

One key advantage of transdermal administration is its ability to bypass first-pass metabolism and factors associated with the gastrointestinal tract, such as pH and the gastric emptying time. Furthermore, it enables sustained and controlled drug release, reduces side effects related to systemic toxicity by minimizing fluctuations in blood concentration, facilitates direct access to the therapeutic target, and enhances treatment adherence, contributing to an overall reduction in therapy costs. Incorporating the drug through nanoparticles aims to improve skin penetration and reduce skin irritation. These nanotechnology-based carriers have shown better performance in dermal delivery compared to conventional systems, facilitating more efficient drug vehiculation in the nanoencapsulated form than in the free form [16,17]. Incorporating FB into nanoencapsulated systems has been explored by other researchers, such as Kim et al. [18], who, in contrast to this study, integrated the drug into lipid nanoparticles. This nanoencapsulation approach provides significant benefits for drug release [Figure 2] [19].

Although nanoparticles offer significant benefits, we also explore incorporating formulations into hydrogels, as hydrogel-based drug delivery systems pose challenges in achieving optimal stability, biodegradability, and targeted efficacy while maintaining biocompatibility. Additionally, hydrogels provide user-friendly administration and convenient storage. Incorporating drugs into hydrogels further enhances the stability and bioavailability of compounds through controlled release mechanisms. Ultimately, this review highlights the potential of hydrogels in biomedical research and underscores the need for continuous innovation to overcome existing challenges and expand their clinical applications [20].

The in vivo efficacy of the formulations was evaluated by performing controlled perforations in mouse ears, simulating the application of an ear tag. Additionally, cytotoxicity and ocular tolerance studies were conducted to assess potential formulation-induced damage and irritability in cases of accidental eye contact, which is particularly relevant for eyebrow piercings. Histological analyses were also performed, and the chemical stability of the formulations was assessed over a three-month period at three different temperatures: 4 °C, 25 °C, and 40 °C.

Both formulations demonstrated enhanced transcutaneous permeability in porcine skin and human skin [8]. Consequently, their anti-inflammatory efficacies were evaluated in both species, along with determining drug retention in porcine skin and human skin.

## 2. Results and Discussion

### 2.1. Effectiveness Studies on Inflammation Prevention

**Figure 3:**
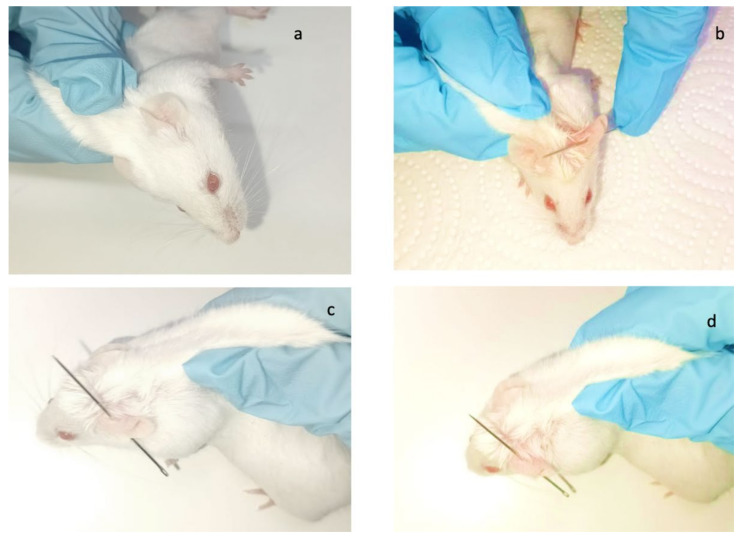
(**a**) Group 1: negative control, without perforation or preventive anti-inflammatory treatment; (**b**) group 2: positive control, which received only perforation; (**c**) group 3: treated with FB-suspension; (**d**) group 4: treated with FB-gel.

To verify that the formulations under study have a preventive effect on reducing inflammation resulting from a perforation process, an in vivo study was conducted, with four groups of mice (*n* = 6) being evaluated in parallel (Figure 3).

**Figure 4:**
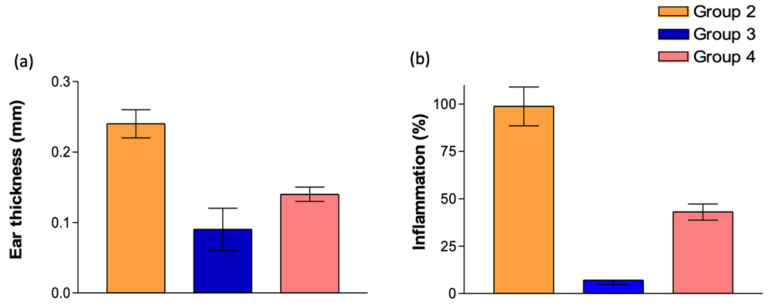
Graphical representations of (**a**) ear thickness and (**b**) percentage of inflammation.

The results shown in Table 1 concern the ear thickness of the rats. Regarding the studied formulations, it can be observed that group 3, corresponding to animals that received FB-suspension, and group 4, corresponding to animals that received FB-gels, exhibited a preventative effect against inflammation, as significant differences were observed among all groups. The graphical representation corresponds to Figure 4a.

After euthanasia was performed on the animals, the ears were weighed, and the percentage of ear inflammation was calculated, considering the weight of the positive control to represent 100% inflammation. On the other hand, since the negative control group did not undergo any induced inflammatory process or receive treatment, the values from this group were deemed not applicable for subsequent statistical analysis.

As indicated in Table 2, significant differences were observed among all groups. Groups 3 and 4, treated with the studied formulations, exhibited inflammation levels below 50% compared to the positive control, demonstrating the efficacy of the preventative action. Furthermore, in both cases, the FB-suspension showed the highest anti-inflammatory effect.

The formulations followed the same activity pattern in both analyzed experiments. Based on ear thickness measurements, statistically significant differences were observed across all groups. These results are also reflected in Figure 4b.

El Moussaoui et al. [21] obtained results similar to ours. They studied inflammation inhibition and found results comparable to those of our study, showing that drugs formulated with nanoparticles in suspension exhibit better anti-inflammatory effects than those formulated in gels.

**Figure 5:**
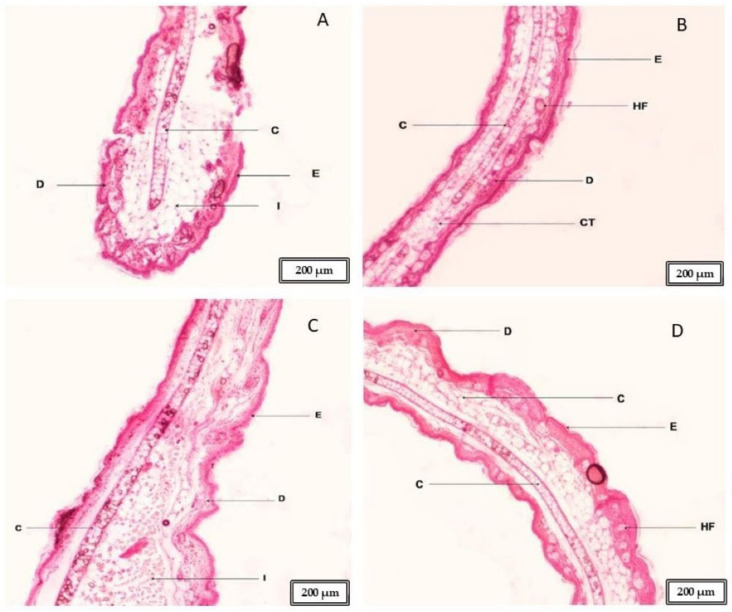
(**A**) Positive control of mouse ear; (**B**) negative control of mouse ear; (**C**) mouse ear where the FB-gel was applied; (**D**) mouse ear where the FB-suspension was applied; E: epidermis; D: dermis; I: inflammation; C: cartilage; CT: connective tissue; HF: hair follicle. Scale bar: 200 μm.

In the mouse ear tissue studies, we observed the positive control (Figure 5A), where the skin is damaged, with a broken epidermis identified. Inflammation in the skin (I) and some inflammatory infiltrates were also observed. This ear corresponds to the mouse that experienced perforation without any treatment. Figure 5B shows the negative control, where the epidermis (E) and dermis (D) are intact and without inflammation. In Figure 5C, we can observe the ear of the mouse to which the gel loaded with flurbiprofen nanoparticles was applied before the piercing. Here, we can see that the formulation improved the inflammation generated by the applied piercing, but not entirely. In the lower zone, we observe an area with inflammatory infiltrates and skin thickening, indicating an inflammatory process (the analyzed skin pieces are sourced from around the piercing site). Finally, in Figure 5D, we see the ear of the mouse where the suspension of flurbiprofen nanoparticles was applied. The skin is structurally well observed, without any inflammation. It is very similar to the negative control, indicating that this formulation prevented inflammation more effectively than the gel studied (this analyzed skin is a piece taken from around the perforation).

Pain and inflammation are among the most common disorders in contemporary clinical medicine, often occurring in processes where piercings are applied [22]. To prevent these inflammatory processes when performing skin piercings, two formulas have been used: a suspension and a gel loaded with flurbiprofen nanoparticles. These formulas were applied in mouse models, specifically in the ears, after piercing with a sterile pin to demonstrate their anti-inflammatory capacities.

In the histological studies of the skin in mouse ears, it was observed that the suspension of flurbiprofen nanoparticles generated more marked and effective prevention of inflammation processes compared to applying the gel, which prevented inflammation, albeit not completely (Figure 5C,D). This is consistent with the permeation studies of both formulations, where more of the flurbiprofen nanoparticle suspension was retained in the skin compared to the gel, which would explain this more marked anti-inflammatory effect in the ears of the mice. It is known that inflammation is an essential protective response of the body, which develops against tissue damage caused by physical trauma, chemical irritants, etc., and to eliminate harmful metabolites and repair tissues. The widespread oral use of NSAIDs such as flurbiprofen has been undertaken, but this can cause some undesirable side effects [23]; thus, nowadays, dermal and transdermal formulations are preferred, as carried out in this study [24].

### 2.2. Cumulative Retained Drug Amount Studies in Porcine and Human Skin (Qr)

The retained amount of FB (Qr) in human and porcine skin from both formulations is determined using Franz cells after administering the formulations topically at 15 and 30 min and subsequently analyzed via HPLC [25]. The objective is to determine the amount of drug retained in the skin of the target species. This assessment is based on applying the product 30 min before perforation or piercing, following the same methodology used to evaluate inflammation prevention.

**Figure 6:**
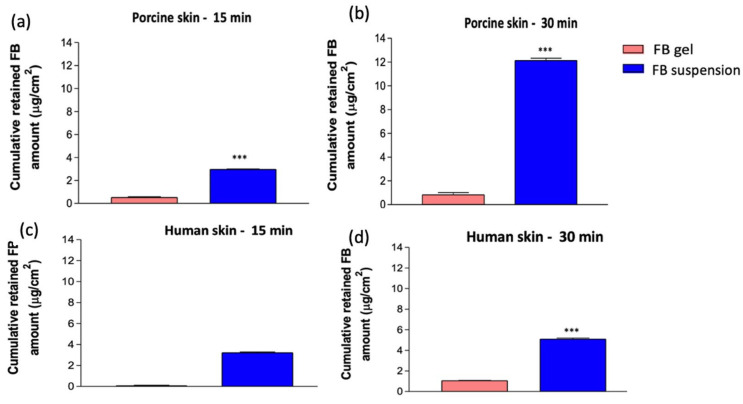
The amount of FB retained in porcine skin (**a**,**b**) and in human skin (**c**,**d**) at 15 and 30 min; ***: *p* < 0.0001.

As shown in Figure 6, Figure 6a,b represent pig skin, while Figure 6c,d correspond to human skin.

Regarding the sampling time, a directly proportional relationship is observed between the duration and the amount retained in both species. At 15 and 30 min, both formulations remain in the susceptible tissues to exert their therapeutic effect. However, from a comparative perspective, the tissue evidently acts as a reservoir, progressively absorbing the active ingredient. This results in a higher amount being retained at 30 min.

Regarding the type of formulation, statistically significant differences are observed, showing that FB incorporated into nanoparticles in suspension is retained much more effectively compared to its delivery via hydrogel. The drug’s ability to be retained by the skin is higher when the FB-suspension is administered in both species. It is well recognized that a drug’s diffusion through a biological membrane is affected not only by its physicochemical traits but also by how the formulation interacts with the skin. Ultimately, when comparing species, porcine skin demonstrates a greater capacity for drug retention after 30 min of the experiment.

Regarding the gel, the amount of drug retained is of the same order, with no significant differences between 15 and 30 min. These results align with those obtained in the previous section, where the inhibition percentage of the FB-suspension is higher than that of the FB-gel. Although the retained amount is lower, this does not exclude the therapeutic efficacy of FB-gel, as seen in the previous section, where both formulations show an efficacy greater than 50% in inflammation inhibition.

### 2.3. Stability Essay

**Figure 7:**
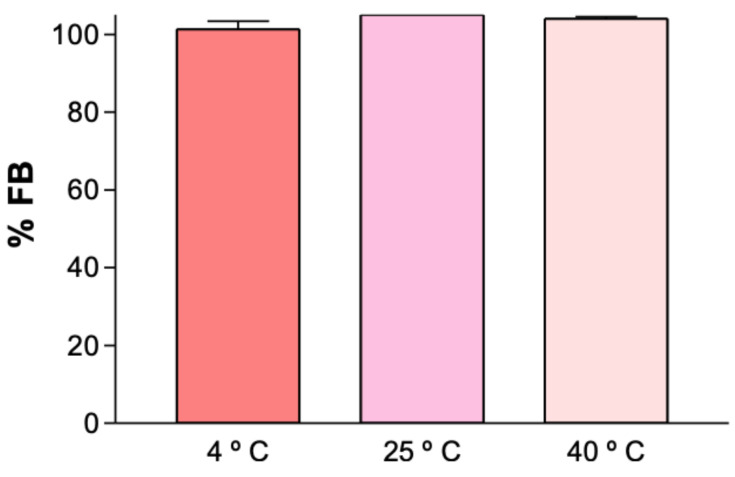
The percentage of FB in the FB-gel formulation stored at different temperatures after three months. No significant differences are observed.

The stability results of the suspensions were published by Ramos et al. [10,11]. Chemical stability studies on the gels were conducted by quantifying the drug content at different temperatures using HPLC analysis to determine the optimal storage temperature range for the formulation. A sample of FB-gel, prepared at room temperature (25 °C), was stored for three months at three different temperatures: 4 °C (refrigerated), 25 °C (ambient), and 40 °C (incubator). At the end of this period, the FB content was assessed using HPLC analysis. The results indicate no decrease in concentration over time, with no statistically significant differences observed in the obtained values (Figure 7).

This stability may be attributed to the presence of the cryoprotectant PEG in the formulation. Studies conducted by Ramos et al. utilized PEG as a cryoprotectant for developing FB nanoparticles intended for ocular administration. It was concluded that formulations containing FB-PEG exhibited superior permeation in human and porcine skin compared to other cryoprotectants [9].

PEG is widely used in biomedical applications, including drug formulations, vaccine stabilization, and regenerative medicine. It is an effective cryoprotectant that prevents ice crystal formation, stabilizes cell membranes and proteins, and reduces osmotic stress during freezing. Its biocompatibility and ability to maintain enzymatic activity make it ideal for the cryopreservation of cells, proteins, and nanoparticles. [26,27]. Gupta et al. reported that incorporating PEG reduced aggregation and facilitated the redispersion of the final product after freezing and dehydration processes [28]. Similarly, other studies have evaluated the effect of PEG on liposomes. For example, Kim et al. [29] demonstrated that PEG-coated liposomes exhibit fewer changes in particle size and reduced aggregate formation during freeze drying, ultimately resulting in enhanced system stability.

It is also important to consider that hydrogels function as highly effective cryoprotectants by regulating temperature fluctuations, inhibiting ice crystal formation, and mitigating thermal stress during freezing and thawing processes. Their polymeric network facilitates water retention, thereby minimizing dehydration at low temperatures, while their viscoelastic properties absorb mechanical stress induced through thermal expansion and contraction [30,31]. Furthermore, hydrogels offer extended thermal stability, creating a protective microenvironment that safeguards cells and biomolecules from extreme temperature variations [32,33].

The FB-gel exhibits stability within a temperature range of 4 °C to 40 °C. However, for user convenience, storage at room temperature (25 °C) is recommended. If presented as a magistral formulation, no specific temperature conditions would be required. As previously mentioned, the excipients accompanying FB are likely responsible for providing thermal protection for three months, consistent with the study period.

### 2.4. In Vitro Cytotoxicity Assesment in HaCaT Cells

**Figure 8:**
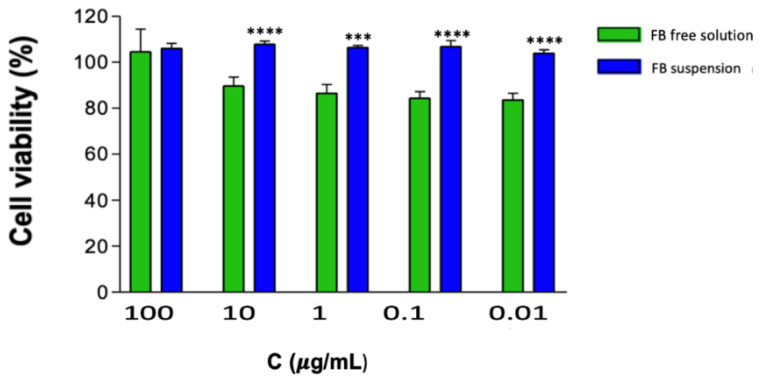
The cell viability of primary human keratinocytes (HaCaT) after 24 h of exposure. The data show significant differences compared to the suspension: *** *p* < 0.0001; **** *p* < 0.00001.

Assessing cell viability is essential for ensuring the safety of the developed formulation and to prevent potential cytotoxic effects that could disrupt skin barriers and compromise the body’s primary protective layer. As shown in Figure 8, the FB-solution does not affect cell viability after 24 h of exposure at any of the tested concentrations. The two formulations shown in Figure 8 exhibit cell viability above 80%, indicating that free FB is not considered cytotoxic. However, the statistically significant differences observed between free FB and FB incorporated into nanoparticles suggest that nanoparticles can reduce cytotoxicity. Therefore, this approach represents a safe and effective drug delivery system. Nanoparticles play a crucial role in reducing drug toxicity by enhancing targeted delivery, controlling release, and improving bioavailability. Encapsulating drugs in nanoparticles minimizes their exposure to healthy tissues, reducing systemic toxicity and side effects. Polymer-based nanoparticles, such as poly(ε-caprolactone), provide sustained drug delivery, decreasing toxicity peaks [34].

FB-NP-mediated drug release may prevent cell death induced by the free drug solution. Since FB is encapsulated with PCL, it reduces the direct contact surface of the active ingredient with HaCat cells, which may explain the statistically significant differences between the two formulations.

In accordance with previous in vitro studies evaluating FB cytotoxicity [35,36], the FB-suspension did not induce cell death rates exceeding 10%, suggesting that it may be safe for dermal administration.

**Figure 9:**
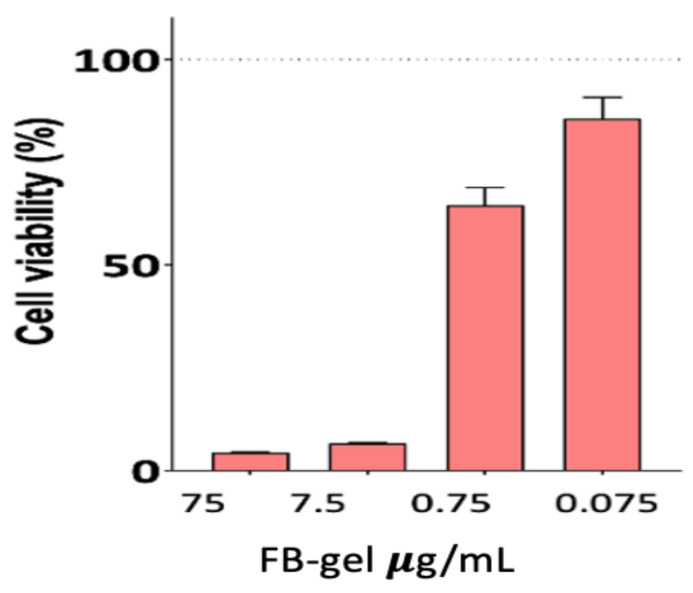
The cell viability of primary human keratinocytes (HaCaT) after 24 h of exposure to the composite gel.

Regarding the gel formulation (Figure 9), a lower cell viability was observed, which improves as the formulation is diluted. However, the gel was not considered toxic, as its cytotoxicity was previously evaluated by Berenguer et al. [37]. They assessed the cytotoxicity of Sepigel^®^ hydrogel on HaCat cells, observing that the inhibitory concentration in the analyzed cell lines was higher than 75 μg/mL, concluding that this excipient is not cytotoxic.

### 2.5. Histological Analysis of Human and Porcine Skin Treated with Different Formulations

**Figure 10:**
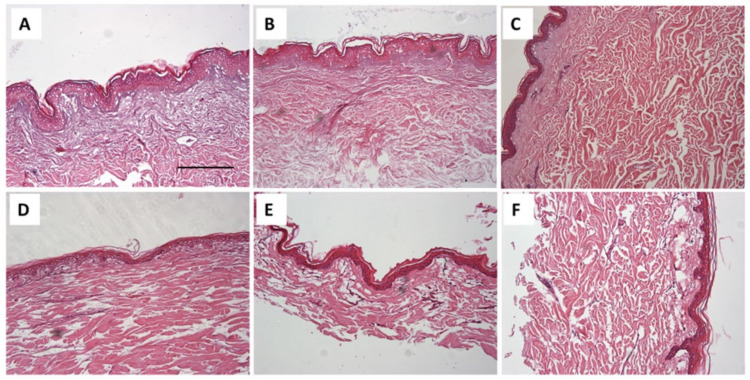
Histological images of human (**A**–**C**) and porcine (**D**–**F**) skin after treatment. Controls (**A**,**D**) show intact structure. FB-gel treated samples (**B**,**E**) maintain human skin integrity but dehydrate porcine skin. FB-suspension (**C**,**F**) causes more dehydration in human skin but better preserves porcine skin. Scale bar = 200 μm.

Histological images (Figure 10) provide a comparative analysis of human (A, B, C) and porcine (D, E, F) skin following treatment with different formulations. Control samples (A, D), treated only with saline solution (SF), exhibit a well-preserved epidermal and dermal structure, serving as a baseline for comparison. In the gel-treated samples (B, E), the stratum corneum in the human skin (B) appears structurally similar to the control (A), whereas porcine skin I shows signs of epidermal dehydration. Conversely, in the suspension-treated samples (C, F), human skin (C) exhibits more pronounced dehydration compared to its control, while porcine skin (F) maintains better hydration relative to the gel-treated group.

These findings suggest that the gel formulation preserves human skin integrity more effectively, whereas the suspension performs better in porcine skin. However, as the treatments were applied prophylactically over a short period, these histological differences are unlikely to significantly impact overall efficacy.

Notably, our previous study [9] evaluated the effect of these formulations on skin barrier function using trans-epidermal water loss (TEWL) measurements in vivo. The results demonstrated that any temporary disruptions in barrier integrity were reversible within a few hours, suggesting that despite the histological differences observed in ex vivo conditions, the formulations do not significantly affect the long-term barrier function of the skin [30,38].

Human skin and porcine skin share several similarities that make them useful in comparative studies, such as the presence of essential lipids—ceramides, cholesterol, and fatty acids—that play key roles in skin barrier function, as well as a generally similar structure composed of the epidermis, dermis, and hypodermis. However, they also present important differences in lipid composition. For example, the specific composition of ceramides varies between the two species, which may influence skin permeability and hydration. Likewise, the proportion and type of fatty acids differ, which can affect the texture of the skin and its response to environmental factors. Pigs have a higher density of sebaceous glands compared to humans, increasing the production of sebum and oilier skin. Moreover, although both types of skin perform a barrier function, there are structural and functional differences that make human skin generally more permeable than porcine skin. Finally, water content also varies, with human skin typically containing a higher amount of water.

Liu et al. [39] point out that the age of pigs is an important factor to consider when comparing species. The skin of young pigs appears to be more similar to that of adult humans than the skin of adult pigs.

On the other hand, as shown in Figure 10, corresponding to the histological analysis section, image E corresponds to porcine skin treated with the FB-gel. We believe that the observed dehydration is due to the gel drawing water from the skin in vitro, where there is no dynamic exchange in the process. Additionally, although the main purpose of PEG-3350 in the formulation is to act as a cryoprotectant agent, its high osmotic capacity should be considered, as this explains its ability to effectively attract and retain water. Since it is a non-absorbable compound, it tends to accumulate in the outermost tissues [40].

### 2.6. In Vitro Tolerance Study: Hen’s Egg Test on the Chorioallantoic Membrane (HET-CAM)

The results for the FB-suspension have already been published by Ramos et al. [11]. Their findings indicate that the FB-suspension does not induce hemorrhage, lysis, or coagulation, suggesting its suitability for periocular application.

**Figure 11:**
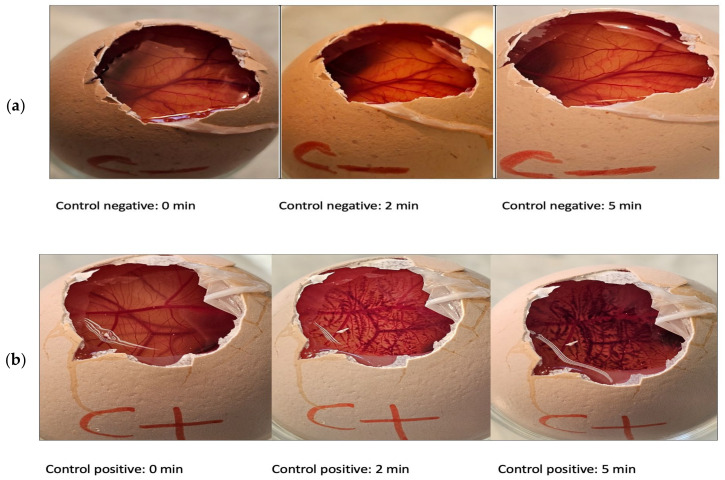

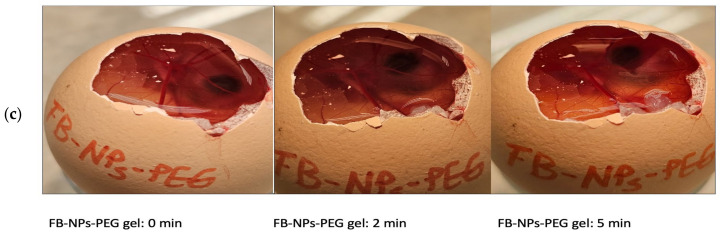
An assessment of the irritant potential of the formulations using the HET-CAM method: (**a**) negative control (saline solution); (**b**) positive control (sodium hydroxide solution 0.1 N; (**c**) FB-gel (study formulation).

As we developed a topical gel formulation for periocular administration, it was essential to evaluate its ocular tolerance. Although our formulations are not intended for direct ocular application, assessing their tolerance was necessary in case of accidental contact with the cornea during periocular piercing. In this context, we conducted an in vitro HET-CAM assay to evaluate the ocular tolerance of the FB-gel formulation. The images corresponding to the experimental process are shown in Figure 11.

The irritation score for the positive control was 20.49 ± 1.95. In contrast, no signs of ocular irritation, such as coagulation, vascular lysis, or hemorrhage, were observed within 5 min when testing the various nanoparticles (including the FB-PEG formulation) and the negative control (0.9% NaCl). The irritancy score remained below 0.1 (Table 3), confirming the safety of the nanoparticle formulations for ocular use, as no reactions were recorded during the test period [41].

In El Bejjaji et al.’s study [9], the NP gel formulation without FB incorporation was evaluated through trans-epidermal water loss (TEWL) in an in vivo experiment with healthy human volunteers to determine whether the excipients in the formulation irritate when administered topically. The TEWL experiment serves as an indirect biomarker of skin irritation, as alterations in the skin barrier are often associated with inflammation, dryness, sensitivity, and increased permeability to irritant substances. It is a key tool for the safety and efficacy assessment of topical products [30,38]. The study concluded that applying the composite gel did not disrupt the stratum corneum and was well tolerated by the skin.

## 3. Conclusions

Our investigation demonstrates the efficacy of flurbiprofen suspension and composite gel formulations in attenuating the inflammation associated with cutaneous perforations, including ear tagging procedures in livestock and periocular piercings or microdermal implants in clinical settings. The FB-suspension exhibited superior anti-inflammatory activity, attributed to enhanced permeability and retention mechanisms. At the same time, the gel formulation offered distinct advantages in terms of application convenience and prolonged storage stability. Both preparations effectively suppressed inflammatory responses, with comprehensive stability and safety assessments confirming their suitability for dermal and periocular applications.

Numerous investigations underscore the need for alternative approaches to minimize distress in animals subjected to ear tagging procedures, as substantial evidence indicates that this practice induces both acute and persistent discomfort. Research consistently demonstrates that ear tagging elicits marked stress responses, emphasizing the necessity for developing less invasive methodologies [3,42]. Analogously, the clinical literature documents vasovagal reactions and anxiety-related manifestations associated with needle phobia in patients undergoing ear piercing. The prophylactic application of these formulations may substantially reduce stress, improve recipient comfort, and facilitate a more conducive procedural environment for practitioners [43].

These results substantiate the therapeutic potential of nanotechnology-based drug delivery systems for managing inflammatory sequelae associated with cutaneous perforations. Future research should focus on refining these formulations for broader dermatological applications while exploring innovative drug delivery mechanisms that enhance bioavailability, improve patient compliance, and, ultimately, lead to better therapeutic outcomes.

In vivo models have demonstrated the pre- and post-procedural anti-inflammatory efficacy of these formulations in mice, and drug retention has been confirmed 30 min post-application in porcine and human skin models.

Accordingly, subsequent research endeavors should emphasize preclinical evaluations using porcine models, given their greater physiological similarity to human integument. Conducting additional preclinical studies in pigs would enable evaluating in vivo kinetics and the impact of the formulation on wound healing. Additionally, its impact on reducing the risk of infection associated with ear tag use can be analyzed, as can its potential contribution to lowering stress levels in animals during this process. These studies would provide valuable information with translational relevance and could help to optimize application protocols.

For prophylactic inflammation management, directly applying liquid formulations to the perforation site prior to intervention warrants further exploration, potentially facilitating incorporation into spray delivery systems [44]. Post-procedural care might benefit from sterile single-dose ampoule packaging to minimize infection risk and optimize healing outcomes.

Alternatively, the gel formulation presents as an optimal candidate, offering practical administration, cost advantages compared to spray systems, and superior stability profiles.

Sepigel^®^ has gained widespread acceptance as a gelling agent in cosmetic preparations, demonstrating remarkable efficacy across diverse pH environments. Beyond its primary thickening function, it confers additional stabilizing and texturizing benefits. Its moderate viscosity characteristics and distinctive opalescent properties contribute to a refreshing, non-occlusive sensory profile, rendering it particularly suitable for dermo-cosmetic applications.

These formulations represent valuable adjunctive therapies for pre- and post-procedurally managing cutaneous perforations, complementing established hygiene protocols designed to prevent infection and enhance wound resolution. While controlled perforations are performed under aseptic conditions, pain and discomfort remain inherent physiological responses to localized tissue trauma. This investigation aims to develop an accessible over-the-counter pharmaceutical preparation addressing these specific requirements in both veterinary and clinical practice.

## 4. Materials and Methods

### 4.1. Materials and Reagents

Flurbiprofen, poly(ε-caprolactone) (Mw ≈ 14,000 g/mol, Mn ≈ 10,000 g/mol, dispersity = 1.4), PEG-3350, and acetone were obtained from Sigma-Aldrich Co. (St. Louis, MO, USA). Poloxamer 188 (P188; Lutrol^®^ F68) was sourced from BASF (Barcelona, Spain), while Sepigel^®^ 305 (polyacrylamide, C13-14 isoparaffin, laureth-7) was procured from Acofarma (Barcelona, Spain). Phosphate-buffered saline (PBS) tablets were purchased from Sigma-Aldrich Chemie (Steinheim, Germany) and prepared per manufacturer’s instructions. Double-distilled water was filtered using a Millipore^®^ system (EMD Millipore, Billerica, MA, USA). High-performance liquid chromatography (HPLC) reagents were obtained from Fisher Scientific (Leicestershire, UK).

### 4.2. Preparation of the Gels Loading Flurbiprofen Nanoparticles

Flurbiprofen nanoparticles were developed using a solvent displacement technique, as described by Fessi et al. [45] and modified by Ramos et al. [10,11]. In total, 15 mg of FB and 49.5 mg of PεCL dissolved in 30 mL of acetone were inserted dropwise into 60 mL of an aqueous P188 solution at a pH of 3.5 under moderate magnetic stirring. The suspension of FB-PεCL was concentrated to 15 mL using a rotary evaporator (R-144; Buchi, Flawil, Switzerland), removing acetone. This process allowed us to obtain an average particle size smaller than 200 nm and a polydispersity index value of 0.088 ± 0.011, common in a monodisperse colloidal suspension, at 25 °C using a Zetasizer Nano ZS (Malvern Instrument, Malvern, UK). Following this line, PEG (160 mg/mL) was added to 15 mL of formulation to protect nanoparticles from stressful processes such as lyophilization.

The nanosuspension (NPs-PEG) was lyophilized, irradiated at 25 KGy, and reconstituted in water. After that, average particle size and the polydispersity index were determined again; they were 187.5 ± 1.5 nm and 0.076 ± 0.013, respectively. In accordance with TEM analysis results, these nanoparticles’ sizes were assayed by El Bejjaji et al. [9].

For the gel formulation, 0.55 g of Sepigel^®^ 305 was added to 5 mL of the nanoparticle suspension under constant agitation, forming a homogenous semisolid nanocomposite gel with a final flurbiprofen concentration of 0.75 mg/mL.

The suspension (NPs-PEG), after being lyophilized and irradiated, was reconstituted in water to prepare two composite gels. To complete the gelation process, 0.55 g of Sepigel^®^ 305 was added to 5 mL of each suspension and mixed thoroughly under agitation to ensure uniform consistency. This resulted in the formation of thin, yet distinct, semisolid composite gel structures [9]. The drug concentration obtained in the final formulation was equivalent to 0.75 mg/mL of flurbiprofen.

### 4.3. Biological Materials

Human and porcine skin were used to determine drug retention in the skin (Qr). This study was approved by the Bioethics Committee of the Barcelona SCIAS Hospital (Protocol Nº002; 17 January 2020). The flank skin of Yorkshire-Landrace pigs was obtained from the animal facility at the Bellvitge Campus of the University of Barcelona (Barcelona, Spain) immediately after the animals were sacrificed for various purposes. The studies were conducted following a protocol approved by the Committee of Animal Experimentation of the Regional Autonomous Government of Catalonia (Spain) and the Animal Experimentation Ethics Committee of the University of Barcelona (Barcelona, Spain) under reference number 7428. Skin samples were sectioned using a GA 630 dermatome (Aesculap, Tuttlingen, Germany) at different thicknesses depending on the skin type—400 and 700 μm for human and porcine skin, respectively, after being frozen at −20 °C.

For in vivo studies, adult male CD-1 mice (20–23 g) were obtained from the Experimental Center for Bioscience at the Faculty of Chemical Sciences and Pharmacy, the National Autonomous University of Honduras (CENBIO-UNAH). The animals were housed in plastic cages with soft bedding, provided with a controlled diet, and given tap water ad libitum. Environmental conditions were maintained at 24 ± 1 °C, with relative humidity between 50 and 60%. Additionally, light conditions followed a 12 h light/12 h dark cycle within each 24 h period. This experiment received approval from the Ethics Committee CENBIO-UNAH (Protocol code CICUAL 002-2025, approved on 14 March 2025).

### 4.4. Drug Analysis and Quantification

**Figure 12:**
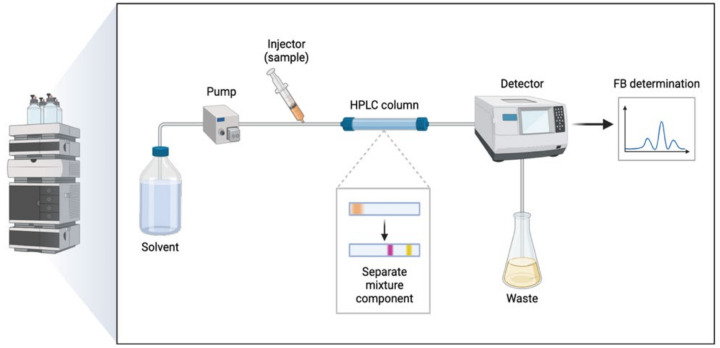
Drug analysis and quantification of flurbiprofen in skin samples using RP-HPLC.

The determination of FB was conducted using high-performance liquid chromatography (HPLC). The conditions are explained in Table 4, and the process is represented in Figure 12.

### 4.5. Methods

#### 4.5.1. Inflammation Prevention Assessment

**Figure 13:**
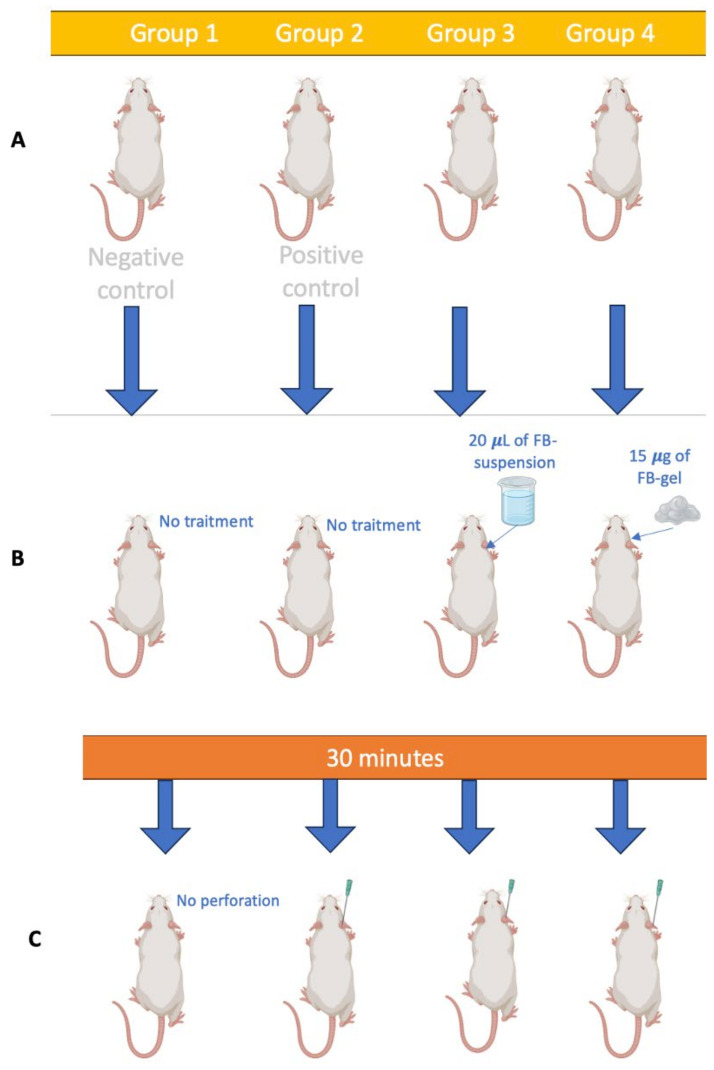
The representation of the in vivo procedure: (**A**) Distribution of the groups; (**B**) the preventative administration of the study formulations to the study groups; (**C**) ear perforation.

To evaluate the in vivo efficacy of the formulations, four groups of male CD-1 mice (n = 6) were formed and kept under standard animal facility conditions according to regulations. The experimental conditions received by each study group are described in Table 5, and the visual representation of the method is detailed in Figure 13.

Groups 3 and 4 received preventative anti-inflammatory treatment topically on the left ear, and after 30 min, perforation was performed on the study ears of groups 2, 3, and 4. Group 1, the negative control, received neither anti-inflammatory treatment nor perforation.

The representation of the procedure is described in Figure 13.

Once the preventative action and the trauma were enacted, the degree of effectiveness was evaluated using two methods. On one hand, the thickness of the ears was measured with a Mitutoyo^®^ 547-561S Thickness Gage Steel digital clipper (Kawasaki, Japan) 45 min after perforation.

**Figure 14:**
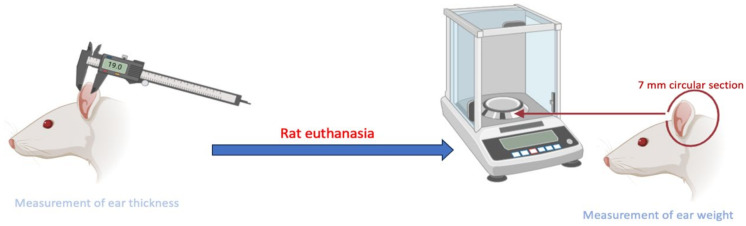
Efficacy analysis process.

Then, after 4 h, the animals were slaughtered, and 7 mm circular sections of the left ear were cut to determine anti-inflammatory activity (Figure 14). The percentage of inhibition was calculated using the following formula:

(1) $$Inflammation\left(\%\right)=\frac{\left(Weight\;\;of\;study\right)}{Weight\;control\;positive}\times 100\;$$

where the weight of the positive control is considered to represent the maximum inflammation (100%), and the weight of the negative control is considered to represent the absence of inflammation (0%), as it did not undergo any process that altered its morphology. As a reference, a 7 mm circular section in the negative control group weighed 0.02 ± 0.01 mg.

#### 4.5.2. Retained Drug Determination

The experiments were conducted in independent vertical Franz diffusion cells with a diffusional surface area of 0.64 cm^2^. Skin tissues were positioned between the two compartments of a Franz cell, with the dermal side in contact with the receptor medium and the epidermal side in contact with the donor chamber. The skin was covered with laboratory film (parafilm, Chicago) to prevent evaporation during this study. Phosphate-buffered saline (PBS) solution at a pH of 7.4 was used as the receptor medium. The permeation study was conducted for 30 min at 32 ± 0.5 °C under continuous stirring in accordance with sink conditions. For the donor compartment, 100 μL of the solution formulation and 125 mg of the gel was applied once the temperature of the skin surface equilibrated to 32 ± 0.5 °C [9]. A saturated solution of FB in PBS was also assayed.

**Figure 15:**
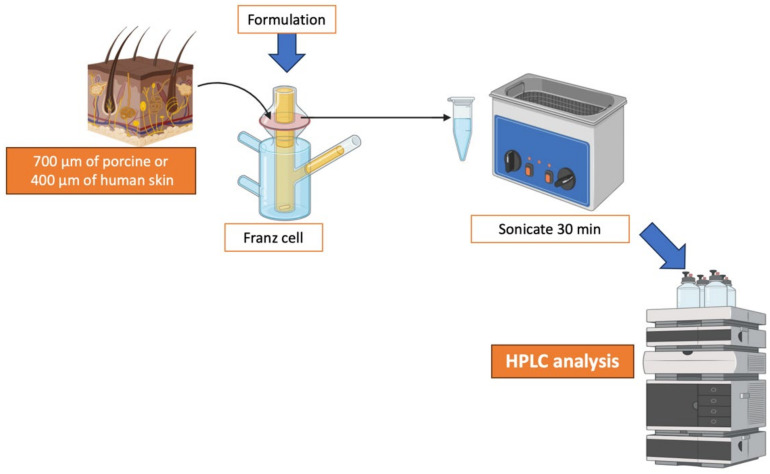
The experimental setup and conditions for the in vitro skin permeation study using Franz diffusion cells.

The porcine and human skin tissues were carefully removed from the Franz cell to determine the retained drug concentration. The skin surface was washed three times with gauze soaked in a 0.05% solution of sodium lauryl sulfate and distilled water. Excess skin surrounding the diffusion area was trimmed, and the exposed surface was gently blotted with filter paper to ensure that it was dry before weighing. The retained FB was extracted using an acetonitrile/water solution (50:50, *v*:*v*) via sonication for 15 min in an ultrasound bath. The resulting solutions were analyzed via RP-HPLC to determine the amount of FB retained in the skin, expressed as Qr (μg/cm^2^). The results were normalized based on the tissue weight and the diffusion area (0.64 cm^2^) and then multiplied by the drug recovery factor. The retained amount of drug in the tissue (Qr, μg/cm^2^) was determined using the following equation:

(2) $$Q_{R}=\frac{(Ex)R}{A\times 100}$$

where *Ex* (μg) is the quantity of drug extracted; *A* (cm^2^) is the effective surface area accessible for diffusion; and R is the drug recovery percentage [46,47]. The experimental conditions are outlined in Table 6 and Figure 15.

#### 4.5.3. Chemical Stability in Different Temperatures

The stability of the suspension was reported by Ramos et al., who concluded that there were no statistically significant differences in stability over the period in which the sample was monitored [10,11].

For the composite gel, stability studies were conducted to determine whether exposure to different temperatures affected the active ingredient. A sample of the composite gel was used as the starting material. For the analysis, 0.1 g of the gel was dissolved in 1 mL of acetonitrile. The samples were stored in Eppendorf tubes at three different temperatures (40 °C, 25 °C, and 4 °C) and analyzed every month for three months. The analysis was performed under validated conditions, as specified in Table 4, with a sample size of n = 5 for each time point.

#### 4.5.4. Cytotoxicity Study in HaCaT Cells

Cell viability in response to the flurbiprofen composite gel and solution was assessed using a 3-(4,5-dimethylthiazol-2-yl)-2,5-diphenyltetrazolium bromide (MTT) assay. The cytotoxicity potential of the free drug was also analyzed.

The immortalized keratinocyte cell line HaCaT was seeded at 2 × 10^5^ cells/mL in 96-well plates (Corning) and incubated at 37 °C with 5% CO₂ for 24 h to allow for cell adhesion. Experiments were conducted once the cell confluence reached 80–90%. HaCaT cells were cultured in Dulbecco’s Modified Eagle’s Medium (DMEM) with high glucose content, supplemented with 25 mM HEPES, 1% non-essential amino acids, 100 U/mL of penicillin, 100 mg/mL of streptomycin, and 10% heat-inactivated fetal bovine serum (FBS).

Various dilutions of the formulations were tested (1/10, 1/100, 1/1000, and 1/10,000).

After 24 h of incubation, the HaCaT cells were washed with 1% sterile PBS and treated with an MTT solution (5 mg/mL) for 2 h at 37 °C. Following this, the medium was carefully aspirated, and 0.1 mL of 99% pure dimethyl sulfoxide (DMSO) was added to lyse the cells and dissolve the purple MTT crystals. The resulting cell lysate was transferred to a fresh 96-well plate, and the absorbance was measured at 540/630 nm excitation/emission wavelengths using an Automatic Microplate Reader (Modulus Microplate Multimode Reader, Turner Biosystems, Sunnyvale, CA, USA).

A negative control consisting of untreated cells was included for comparison. Absorbance values were directly proportional to cell viability, and the percentage of cell viability was calculated using the following equation:

(3) $$Cell\;viability=\frac{ABS\;treated\;cells}{ABS\;control\;cells}\times 100$$

The experiment was conducted in parallel since the two formulations had different concentrations. The suspension and free drug were tested at a concentration of 1 mg/mL (Figure 8), whereas the composite gel was formulated at 0.75 mg/mL (Figure 9).

#### 4.5.5. Histological Analysis of Mouse Ear Tissue Following Perforation

For fixing the samples, several pieces cut from the treated mouse ears, positive control, and negative control were immersed for 24 h in a fixative mixture called Orth-ER liquid, which is a combination of potassium dichromate (5 g), glacial acetic acid (5 mL), commercial formalin (5 mL) (MERK, Darmstadt, Germany), and distilled water (90 mL). This solution must remain protected from light and heat. Next, the excess fixative was washed off with constant running water for 4 h. To dehydrate without causing damage to the tissues, the samples were immersed in a gradual series of ethanol solutions at different concentrations (50%, 60%, 70%, 80%, 90%, 95%, and 99%) for an average of 6 h at each concentration (DIMELAB, Tegucigalpa, Honduras).

The tissue had to be de-alcoholized, for which a lightening substance was used, making the tissue transparent and soluble in kerosene, where the sample to be cut was placed. In this case, Xylol (DIMELAB, Tegucigalpa, Honduras) was used for 6 h. Introducing the tissues in the kerosene had to be gradual, so the tissue was introduced in liquid kerosene of three parts xylol and one part kerosene (Química Industrial, Tegucigalpa, Honduras), then, in equal parts, one part solvent and three parts kerosene, ending with 100% kerosene; these changes were made at a temperature of 56 to 60 °C, and each change took, on average, from 3 to 6 h. The definitive inclusion was performed using metallic lead bars to position the tissue in the plane to be cut (transverse cut). Once the kerosene with the tissue was solidified at room temperature, the wooden block was placed as a support to perform the cuts. A Minot-type microtome (AO Scientific Instruments) was used to make the cuts, cutting the tissues in a thickness of 10 μm and performing kerosene stretching in water baths with thermal regulation. The cut sections were placed on a slide previously treated with an adhesive solution (previously prepared Haupt glue). For tissue staining, the hematoxylin/eosin combination staining battery technique (MERK, Darmstadt, Germany) was used. Finally, the coverslip was cleaned and glued with Entellan resin (MERK, Darmstadt, Germany), placed in an oven at a temperature of 28 to 35 °C, and left to cool for at least 24 h. It was then viewed and analyzed under an Olympus CX31 microscope equipped with a camera. The results are shown in Figure 5.

#### 4.5.6. Histological Analysis of Porcine and Human Skin

Skin samples were processed using standard histological techniques. The samples were fixed in 10% neutral-buffered formalin at room temperature for 24 h, followed by dehydration through a graded ethanol series, clearing in xylene, and embedding in paraffin. Thin sections of 5 μm thickness were obtained using a rotary microtome and stained with hematoxylin and eosin (H&E) for microscopic evaluation.

The stained sections were examined under an Olympus BX41 light microscope equipped with an Olympus XC50 digital camera (Olympus Co., Tokyo, Japan) to assess tissue morphology and structural integrity. Images were captured and analyzed to identify any histopathological alterations. Quantitative evaluations were performed using ImageJ software, version 1.54k (NIH, Bethesda, MD, USA) in a blinded manner to ensure objectivity. The results correspond to Figure 10.

#### 4.5.7. In Vitro Tolerance Study: Hen’s Egg Test on the Chorioallantoic Membrane (HET-CAM)

The potential ocular irritation of the flurbiprofen composite gel was assessed using the hen’s egg test on the chorioallantoic membrane (HET-CAM). This in vitro test evaluated toxicity by observing its effects on the chorioallantoic membrane (CAM) of 10-day-old embryonated hen’s eggs, sourced from the G.A.L.L.S.A. farm, Tarragona, Spain. The reactions were monitored at two time points, 2 min and 5 min, within a 5 min observation window, focusing on the onset of hemorrhage (bleeding), coagulation (disintegration of blood vessels), and vessel lysis (protein denaturation both within and outside of blood vessels) [48].

Each response was analyzed separately, and the irritation score (IS) was calculated by combining the individual effects to classify the irritancy level of the substance. The irritation score was determined using the following equation:

(4) $$IS=\frac{301-H}{300}\times 5+\frac{301-L}{300}\times 7+\frac{301-C}{300}\times 9$$

where *H* stands for hemorrhage; *L* stands for vessel lysis; *C* stands for coagulation; and time (s) stands for the number of seconds after which each reaction was observed. A total of 300 μL of the test substance was applied to the CAM, and the membrane was observed for 2 and 5 min to assess the severity of each reaction in accordance with the INVITTOX protocol [49,50]. NaOH (0.1 N) was used as the positive control, while a 0.9% NaCl solution served as the negative control. The interpretation of the results from Equation (4) can be derived from Table 7.

### 4.6. Statistical Analysis

The results are reported as mean ± standard deviation. Statistical differences were determined using one-way analysis of variance (ANOVA) in GraphPad Prism^®^ software v. 5.0 (GraphPad Software Inc., San Diego, CA, USA).

For in vivo efficacy and stability studies, the Tukey post hoc test was applied, while Student’s *t*-test was used to assess cytotoxicity, as shown in Figure 8. A *p*-value < 0.05 was considered statistically significant.

## Figures and Tables

**Table 1:** Measurements of ear thickness.

	Thickness (mm)	*p* Value
Group 1	0.06 ± 0.05	Not applicable
Group 2	0.24 ± 0.02	*p* < 0.001
Group 3	0.09 ± 0.03 ^a^	*p* < 0.001
Group 4	0.14 ± 0.01 ^a,b^	*p* < 0.001

^a^ Statistically significant difference compared to group 2; ^b^ statistically significant difference compared to group 3.

**Table 2:** Inflammation percentage.

	Inflammation (%)	*p* Value
Group 1	Not applicable	Not applicable
Group 2	98.74 ± 10.2 ^a^	*p* < 0.001
Group 3	6.98 ± 2.24 ^a^	*p* < 0.001
Group 4	43.02 ± 4.25 ^a,b^	*p* < 0.01

^a^ Statistically significant difference compared to group 2; ^b^ statistically significant difference compared to group 3.

**Table 3:** Irritation score (IS) of the composite gel tested using the HET-CAM technique.

Formulation	Irritation Score (IS)
FB-gel	0.03

**Table 4:** The chromatographic conditions for the determination of flurbiprofen.

Parameter	Conditions
Chromatographic column	Luna ^®^ 5 μm C18 (2) 100 Å (150 × 4.6 mm)
Mobile phase	Acetonitrile: PBS (pH 2.5) (65:25) (*v*/*v*)
Flux	1.5 mL/min
Injection volume for Qr	10 μL
Run time	3.2 min
Wavelength	245 nm
Stability range	400–0.391 μg/mL
Qr range	100–6.25 μg/mL

**Table 5:** The classification of the groups.

	Preventive Treatment	Perforation	
Group 1	No	No	Negative control
Group 2	No	Yes	Positive control
Group 3	FB-suspension	Yes	Study group
Group 4	FB-gel	Yes	Study group

**Table 6:** The experimental conditions for the ex vivo permeation test (*Qret*).

Parameter	Conditions
Receptor fluid	PBS (pH 7.4)
Cell volume	6 mL
Diffusion area	0.64 cm^2^
Membrane	Pig and human skin
Replicates	5 replicates
Temperature	32 ± 0.5 °C
Stirring	500 r.p.m.
Dose	1 mg/mL
Sample volume (solution)	100 μL
Sample weight (gel)	125 mg
Sampling times	0 (pre- sampling), 15 min, 30 min

**Table 7:** Classification parameters for irritation severity.

Irritation Measurement	Severity Categorization
0–0.9	Non-Irritant
1–4.9	Slight Irritant
5–8.9	Moderate Irritant
9–21	Severe Irritant

## Data Availability

The data presented in this study are available upon request from the corresponding author. As the data are part of a doctoral thesis, they will not be available until the thesis has been published.

## References

[1] Caja G., Hernández-Jover M., Conill C., Garín D., Alabern X., Farriol B., Ghirardi J. (2005). Use of ear tags and injectable transponders for the identification and traceability of pigs from birth to the end of the slaughter line. J. Anim. Sci..

[2] Harmon M.L., Downey B.C., Drwencke A.M., Tucker C.B. (2023). Development and Application of a Novel Approach to Scoring Ear Tag Wounds in Dairy Calves. J. Dairy Sci..

[3] Barz A., Ritzmann M., Breitinger I., Langhoff R., Zols S., Palzer A., Heinritzi K. (2010). Examination of different options for combined administration of an NSAID (meloxicam) and iron for piglets being castrated. Tierärztliche Praxis. Ausg. G Grosstiere/Nutztiere.

[4] Hayer J.J., Nysar D., Schmitz A., Leubner C.D., Heinemann C., Steinhoff-Wagner J. (2022). Wound lesions caused by ear tagging in unweaned calves: Assessing the prevalence of wound lesions and identifying risk factors. Animmal.

[5] Malik M., Chiers K., Theuns S., Vereecke N., Chantziaras I., Croubels S., Maes D. (2023). Porcine ear necrosis: Characterization of lesions and associated pathogens. Vet. Res..

[6] Numberger J., Ritzmann M., Übel N., Eddicks M., Reese S., Zöls S. (2016). Ear tagging in piglets: The cortisol response with and without analgesia in comparison with castration and tail docking. Animal.

[7] Van Hoover C., Rademayer C.A., Farley C.L. (2017). Body Piercing: Motivations and Implications for Health. J. Midwifery Womens Health.

[8] Sheldon R.R., Loughren M.J., Marenco C.W., Winters J.R., Bingham J.R., Martin M.J., Eckert M.J., Burney R.O. (2019). Microdermal Implants Show No Effect on Surrounding Tissue During Surgery With Electrocautery. J. Surg. Res..

[9] El Bejjaji S., Ramos-Yacasi G., Suñer-Carbó J., Mallandrich M., Goršek L., Quilchez C., Calpena A.C. (2024). Nanocomposite Gels Loaded with Flurbiprofen: Characterization and Skin Permeability Assessment in Different Skin Species. Gels.

[10] Ramos-Yacasi G.R., Calpena-Campmany A.C., Egea-Gras M.A., Espina-García M., García-López M.L. (2017). Freeze Drying Optimization of Polymeric Nanoparticles for Ocular Flurbiprofen Delivery: Effect of Protectant Agents and Critical Process Parameters on Long-Term Stability. Drug Dev. Ind. Pharm..

[11] Ramos Yacasi G.R., García Lopéz M.L., Espina García M., Parra Coca A., Calpena Campmany A.C. (2016). The Influence of Freeze Drying and Gamma-Irradiation in Pre-Clinical Studies of Flurbiprofen Polymeric Nanoparticles for Ocular Delivery Using D-(+)-Trehalose and Polyethylene Glycol. Int. J. Nanomed..

[12] Oscanoa-Espinoza T., Lizaraso-Soto F. (2015). Antiinflamatorios No Esteroides: Seguridad Gastrointestinal, Cardiovascular y Renal. Rev. Gastroenterol..

[13] Salido A., Abásolo M., Bañares L. (2001). Revisión de los antiinflamatorios inhibidores selectivos de la ciclooxigenasa-2. Inf. Ter. Sist. Nac. Salud.

[14] Dorbandt D.M., Labelle A.L., Mitchell M.A., Hamor R.E. (2017). The Effects of Topical Diclofenac, Topical Flurbiprofen, and Humidity on Corneal Sensitivity in Normal Dogs. Vet. Ophthalmol..

[15] Roberts B.M., Geddis A.V., Matheny R.W. (2024). The dose-response effects of flurbiprofen, indomethacin, ibuprofen, and naproxen on primary skeletal muscle cells. J. Int. Soc. Sports Nutr..

[16] Siafaka P.I., Özcan Bülbül E., Okur M.E., Karantas I.D., Üstündağ Okur N. (2023). The Application of Nanogels as Efficient Drug Delivery Platforms for Dermal/Transdermal Delivery. Gels.

[17] Berthet M., Gauthier Y., Lacroix C., Verrier B., Monge C. (2017). Nanoparticle-Based Dressing: The Future of Wound Treatment?. Trends Biotechnol..

[18] Kim R.M., Jang D.-J., Kim Y.C., Yoon J.-H., Min K.A., Maeng H.-J., Cho K.H. (2018). Flurbiprofen-Loaded Solid SNEDDS Preconcentrate for the Enhanced Solubility, In-Vitro Dissolution and Bioavailability in Rats. Pharmaceutics.

[19] Alves G.L., Teixeira F.V., da Rocha P.B.R., Krawczyk-Santos A.P., Andrade L.M., Cunha-Filho M., Marreto R.N., Taveira S.F. (2022). Preformulation and characterization of raloxifene-loaded lipid nanoparticles for transdermal administration. Drug Deliv. Transl. Res..

[20] Liu B., Chen K. (2024). Advances in Hydrogel-Based Drug Delivery Systems. Gels.

[21] El Moussaoui S., Abo-Horan I., Halbaut L., Alonso C., Coderch L., Garduño-Ramírez M.L., Clares B., Soriano J.L., Calpena A.C., Fernández-Campos F. (2021). Polymeric Nanoparticles and Chitosan Gel Loading Ketorolac Tromethamine to Alleviate Pain Associated with Condyloma Acuminata during the Pre- and Post-Ablation. Pharmaceutics.

[22] Oktay A.N., Tamer S.I., Han S., Uludag O., Celebi N. (2020). Preparation and In Vitro/In Vivo Evaluation of Flurbiprofen Nanosuspension-Based Gel for Dermal Application. Eur. J. Pharm. Sci..

[23] Pireddu R., Caddeo C., Valenti D., Marongiu F., Scano A., Ennas G., Sinico C. (2016). Diclofenac acid nanocrystals as an effective strategy to reduce in vivo skin inflammation by improving dermal drug bioavailability. Colloids Surf. B Biointerfaces.

[24] Kawadkar J., Pathak A., Kishore R., Chauhan M.K. (2012). Formulation, characterization and in vitro–in vivo evaluation of flurbiprofen-loaded nanostructured lipid carriers for transdermal delivery. Drug Dev. Ind. Pharm..

[25] Parra A., Clares B., Rosselló A., Garduño-Ramírez M.L., Abrego G., García M.L., Calpena A.C. (2016). Ex vivo permeation of carprofen from nanoparticles: A comprehensive study through human, porcine and bovine skin as anti-inflammatory agent. Int. J. Pharm..

[26] Zhou C., Sun M., Wang D., Yang M., Loh J.L.C., Xu Y., Zhang R. (2024). In vitro antibacterial and anti-inflammatory properties of imidazolium poly (ionic liquids) microspheres loaded in GelMA-PEG hydrogels. Gels.

[27] Thangavelu M., Kim P.-Y., Cho H., Song J.-E., Park S., Bucciarelli A., Khang G. (2024). A gellan gum, polyethylene glycol, hydroxyapatite composite scaffold with the addition of ginseng-derived compound K with possible applications in bone regeneration. Gels.

[28] Gupta A., Smith B., Kumar C., Lee D., Johnson E. (2012). Evaluation of PEGylation as a cryoprotectant in PLGA nanoparticles. J. Control. Release.

[29] Kim S., Park J., Lee Y., Choi H., Cho S. (2014). Enhancement of Liposomal Stability by PEGylation: Effects on Cryoprotection and Redispersibility. Int. J. Pharm..

[30] Chirikhina E., Chirikhin A., Xiao P., Dewsbury-Ennis S., Bianconi F. (2020). In vivo assessment of water content, trans-epidermal water loss and thickness in human facial skin. Appl. Sci..

[31] Lin C.-C. (2015). Recent advances in crosslinking chemistry of biomimetic poly (ethylene glycol) hydrogels. RSC Adv..

[32] Wang X., Xu H. (2010). Incorporation of DMSO and dextran-40 into a gelatin/alginate hydrogel for controlled assembled cell cryopreservation. Cryobiology.

[33] Zhang C., Zhou Y., Zhang L., Wu L., Chen Y., Xie D., Chen W. (2018). Hydrogel cryopreservation system: An effective method for cell storage. Int. J. Mol. Sci..

[34] Beirampour N., Bustos-Salgado P., Garrós N., Mohammadi-Meyabadi R., Domènech Ò., Suñer-Carbó J., Rodríguez-Lagunas M.J., Kapravelou G., Montes M.J., Calpena A. (2024). Formulation of Polymeric Nanoparticles Loading Baricitinib as a Topical Approach in Ocular Application. Pharmaceutics.

[35] Tanino T., Funakami Y., Nagai N., Kato Y. (2015). Cyclosporin A-sensitive cytotoxicity of flurbiprofen non-stereoselectively mediated by cytochrome P450 metabolism in three-dimensional cultured rat hepatocytes. J. Pharm. Pharmacol..

[36] Wang L., Bao S.H., Pan P.P., Xia M.M., Chen M.C., Liang B.Q., Dai D.P., Cai J.P., Hu G.X. (2015). Effect of CYP2C9 genetic polymorphism on the metabolism of flurbiprofen in vitro. Drug Dev. Ind. Pharm..

[37] Berenguer D., Alcover M.M., Sessa M., Halbaut L., Guillén C., Boix-Montañés A., Fisa R., Calpena-Campmany A.C., Riera C., Sosa L. (2020). Topical Amphotericin B Semisolid Dosage Form for Cutaneous Leishmaniasis: Physicochemical Characterization, Ex Vivo Skin Permeation and Biological Activity. Pharmaceutics.

[38] Rauscher M., Rauscher A., Hu L.Y., Schlitt H.J., Krauß S., Illg C., Reis Wolfertstetter P., Hofmann A., Knorr C., Denzinger M. (2024). Influence of Accumulation of Humidity under Wound Dressings and Effects on Transepidermal Water Loss (TEWL) and Skin Hydration. Appl. Sci..

[39] Liu Y., Chen J.Y., Shang H.T., Liu C.E., Wang Y., Niu R., Wei H. (2010). Light microscopic, electron microscopic, and immunohisto- chemical comparison of Bama minipig (Sus scrofa domestica) and human skin. Comp. Med..

[40] Schiller L.R., Emmett M., Santa Ana C.A., Fordtran J.S. (1988). Osmotic effects of polyethylene glycol. Gastroenterology.

[41] Avram Ș., Bora L., Vlaia L.L., Muț A.M., Olteanu G.-E., Olariu I., Magyari-Pavel I.Z., Minda D., Diaconeasa Z., Sfirloaga P. (2023). Cutaneous Polymeric-Micelles-Based Hydrogel Containing *Origanum vulgare* L. Essential Oil: In Vitro Release and Permeation, Angiogenesis, and Safety Profile In Ovo. Pharmaceuticals.

[42] Sutherland M.A., Davis B.L., Brooks T.A., Coetzee J.F. (2012). The Physiological and Behavioral Response of Pigs Castrated with and without Anesthesia or Analgesia. J. Anim. Sci..

[43] Xie Z.A., Zhang K.L., Han F., Tang M.Y., Chen J.W., Liu G.P. (2023). The Incidence of Vasovagal Reactions during Earlobe Piercing. Front. Med. (Lausanne).

[44] Angsusing J., Samee W., Tadtong S., Mangmool S., Okonogi S., Toolmal N., Chittasupho C. (2025). Development, Optimization, and Stability Study of a Yataprasen Film-Forming Spray for Musculoskeletal Pain Management. Gels.

[45] Fessi H., Puisieux F., Devissaguet J.P., Ammoury N., Benita S. (1989). Nanocapsule formation by interfacial polymer deposition following solvent displacement. Int. J. Pharm..

[46] Bouwstra J.A., Honeywell-Nguye P.L. (2002). Skin structure and mode of action of vesicles. Adv. Drug Deliv. Rev..

[47] Ferrero C., Massuelle D., Doelker E. (2010). Towards elucidation of the drug release mechanism from compressed hydrophilic matrices made of cellulose ethers. II. Evaluation of a possible swelling-controlled drug release mechanism using dimensionless analysis. J. Control Release..

[48] Lorenzo-Veiga B., Diaz-Rodriguez P., Alvarez-Lorenzo C., Loftsson T., Sigurdsson H.H. (2020). In Vitro and Ex Vivo Evaluation of Nepafenac-Based Cyclodextrin Microparticles for Treatment of Eye Inflammation. Nanomaterials.

[49] Interagency Coordinating Committee on the Validation of Alternative Methods (ICCVAM) (2010). Test Method Protocol: Hen’s Egg Test-Chorioallantoic Membrane (HET-CAM) Test Method.

[50] Spielmann H. (1995). HET-CAM Test. Methods Mol. Biol..

